# Identification of Genes Potentially Associated with the Fertility Instability of S-Type Cytoplasmic Male Sterility in Maize via Bulked Segregant RNA-Seq

**DOI:** 10.1371/journal.pone.0163489

**Published:** 2016-09-26

**Authors:** Aiguo Su, Wei Song, Jinfeng Xing, Yanxin Zhao, Ruyang Zhang, Chunhui Li, Minxiao Duan, Meijie Luo, Zi Shi, Jiuran Zhao

**Affiliations:** Maize Research Center, Beijing Academy of Agriculture and Forestry Sciences, Beijing Key Laboratory of Maize DNA Fingerprinting and Molecular Breeding, Beijing, 100097, China; Saint Mary's University, CANADA

## Abstract

S-type cytoplasmic male sterility (CMS-S) is the largest group among the three major types of CMS in maize. CMS-S exhibits fertility instability as a partial fertility restoration in a specific nuclear genetic background, which impedes its commercial application in hybrid breeding programs. The fertility instability phenomenon of CMS-S is controlled by several minor quantitative trait locus (QTLs), but not the major nuclear fertility restorer (*Rf3*). However, the gene mapping of these minor QTLs and the molecular mechanism of the genetic modifications are still unclear. Using completely sterile and partially rescued plants of fertility instable line (FIL)-B, we performed bulk segregant RNA-Seq and identified six potential associated genes in minor effect QTLs contributing to fertility instability. Analyses demonstrate that these potential associated genes may be involved in biological processes, such as floral organ differentiation and development regulation, energy metabolism and carbohydrates biosynthesis, which results in a partial anther exsertion and pollen fertility restoration in the partially rescued plants. The single nucleotide polymorphisms (SNPs) identified in two potential associated genes were validated to be related to the fertility restoration phenotype by KASP marker assays. This novel knowledge contributes to the understanding of the molecular mechanism of the partial fertility restoration of CMS-S in maize and thus helps to guide the breeding programs.

## Introduction

Cytoplasmic male sterility (CMS) is a common phenomenon in higher plants and is characterized by maternal inheritance, pollen sterility and normal pistil development [1]. Plant CMS is mediated by nuclear-mitochondrial interactions in which the sterility results from the expression of mitochondrial genes and can be restored by nuclear restoring fertility (*Rf*) genes [2–4]. The CMS system is widely applied in crop hybrid breeding to avoid extra efforts for artificial emasculation [5]. Therefore, CMS has important theoretical and commercial value in hybrid seed production. Maize (*Zea mays* L.) has three major types of CMS, designated as T (Texas), C (Charrua) and S (USDA). The S-type of CMS (CMS-S) is the largest group among the three types and has wide cytoplasmic sources [6]. Unfortunately, S-type is also the most fertility instable type and exhibits incomplete male sterility in sterile lines under specific genetic backgrounds [7, 8]. This fertility instability requires careful supervision in the field and therefore negatively affects the commercial application of CMS-S in maize hybrid breeding.

In maize CMS-S, a 1.6-kb transcript of the mitochondrial genome R region, containing the chimeric gene sequence *orf355-orf77*, contributes to pollen sterility during pollen development [9, 10], while the nuclear fertility restorer *Rf3* affects its transcription, resulting in fertility restoration in the sterile lines[11, 12]. In addition, the fertility restoration of CMS-S is mainly controlled by the major restorer *Rf3*, which is mapped to chromosome 2 within bin2.09 [13, 14].

The fertility instability of CMS-S is characterized by the appearance of partially rescued plants in the offspring of sterile lines, in which the pollen becomes partially viable. Fertility instability deviates under specific nuclear genetic backgrounds [15]. The ratios of instability of CMS-S are observed at a relatively high level with Wf9 and M825 nuclear genomes [16]. Previous research indicates that multiple genes might be involved in the fertility instability of CMS-S. Recently, Feng et al. identified 30 significant loci for pollen fertility, anther exsertion and pollen shedding in CMS-S maize using a mapping panel of 513 plants in three environments, suggesting that in addition to the major restorer *Rf3*, multiple minor genetic loci are involved in fertility instability [17]. In addition, Tie et al. detected 6 quantitative trait locus (QTLs) with significant effects on the male fertility of CMS-S [18], and Kohls et al. also identified 7 QTLs for the partial fertility restoration of CMS-C [19]. Moreover, the expression of another restoring gene, *Rf9*, which is a less effective fertility restorer compared to *Rf3*, is influenced by both nuclear background and temperature [20]. Nevertheless, the molecular mechanism of the fertility instability of CMS-S is still unknown because this phenomenon is considerably complicated and can be easily influenced by many environmental factors.

Bulk segregant RNA-Seq (BSR) is a modification of bulked segregant analysis (BSA) and utilizes the RNA-seq reads to map genes in a rapid and efficient manner [21, 22]. BSR-Seq provides not only the position of the target genes but also the differential expression pattern of potential associated genes between the bulks. It can also facilitate in *de novo* SNP discovery for marker development in breeding programs. Thus, BSR-Seq has been successfully utilized in maize genetic mapping for the accumulation of epicuticular waxes, oil biosynthesis and other traits [21, 23, 24].

The maize inbred line Jing724 is the maternal parent of Jingke968, which is one of the leading varieties in China. Fertility instable line (FIL)-B was obtained using marker-assisted selection by backcrossing the CMS-S sterile line MD32, which possesses the desired agronomic traits and complete pollen sterility, to Jing724 [25, 26]. In the BC_3_ population, FIL-B showed partial fertility restoration, exhibiting anther exsertion with no anther dehiscence or pollen shedding. Thus, understanding the genetic architecture of the fertility instability in CMS-S is crucial for the preferably application of the three-line breeding in maize.

The objectives of this study were to (1) determine the genomic regions containing minor genetic loci associated with partial fertility restoration; (2) predict the potential associated genes involved in fertility instability using the available genomic resources; and (3) examine their differential expression patterns in anthers and validate the SNPs identified from RNA-Seq.

## Materials and Methods

### Plant materials

Jing724 is an inbred line that was developed and released by the Maize Research Center, Beijing Academy of Agricultural and Forestry Science. By crossing to the CMS-S sterile line MD32, the sterile F_1_ was obtained, which was consecutively backcrossed to Jing724. A few families of the BC_3_ population exhibited partial fertility instability with anther exsertion. Molecular markers were applied to select the Jing724 nuclear background in each backcross generation (S4 Table), which made the sterile and partially rescued families as near isogenic lines. The BC_4_ population was acquired from a backcross of sterile male individuals from the fertility segregating family in the BC_3_ to inbred Jing724, which was designated as FIL-B. The plant materials of the inbred line Jing724 and FIL-B were planted in the Hainan Maize Propagation Base in Yacheng, Hainan (HN-YC, 18.3°N, 109.5°E) for sample collection.

### Identification of fertility

Anther exsertion and anther dehiscence were observed for individual plants in the field from the start of the tasseling stage to the end of the pollinating stage. Three spikelets, respectively positioned at the top, in the middle and at the bottom of the tassel, were collected from each of the 30 plants of both completely sterile plants and plants with partial anther exsertion. The spikelets were then stored in Carony’s solution [75% (v/v) ethanol in acetic acid] in 1.5 ml eppendorf tubes. The pollen was stained and scored as previously described by Zhu et al. [27] and was examined using a light microscope (Olympus IX73, Japan) at 40 × magnification. Three representative fields of each sample were used to calculate the proportion of viable pollen in the sterile plants and the partially rescued plants.

### Pollen microspore development

Spikelets of FIL-B were sampled every three days for the whole course of tassel development. The samples were fixed in Carony's solution and stored in 70% ethanol. The anther samples were dehydrated, embedded and sliced as previously described by Zhu et al. [27]. The slices were stained with safranin-green and sealed in neutral gum after toasting at 60°C. The images were obtained using an Olympus IX73 microscope (Olympus, Tokyo) at 100 × magnification a week after drying.

### Analysis of genes with differential expression

The anthers of the completely sterile and partially rescued plants were collected from 30 biological replicates and stored in a 10x volume RNA wait solution (Solarbio, Beijing). The RNA was extracted by Trizol, and the integrity and concentration were determined by Agilent2010 (Agilent, Santa Clara, CA) to ensure that the RIN value was greater than 6.8. Thirty RNA samples from the partially rescued plants were equally mixed and designated as bulk F, while 30 samples from the sterile plants were mixed and designated bulk S. The transcriptomes of both bulks were sequenced at Biomarker Technologies Co., LTD (Beijing, China) using Illumina HiSeq2500 (Illumina, San Diego, CA).

The clean data were aligned to a maize reference genome (B73 RefGen_v3 ftp://ftp.ensemblgenomes.org/pub/plants/release-24/fasta/) to create the mapped data. The differentially expressed genes (Fold change ≥2 and FDR<0.01) were identified by EBSeq [28]. Multiple databases were utilized to annotate the differentially expressed genes, including NR[29], Swiss-Prot[30], GO[31], COG[32], and KEGG[33].

### QTLs mapping by BSR-Seq

The sequencing reads of both bulks were aligned to the reference genome by STAR (2.3.0e, https://github.com/alexdobin/STAR/releases), and the SNPs were identified by GATK (3.1–1, https://www.broadinstitute.org/gatk/index.php). Because the frequencies cannot be accurately determined at low read counts, a minimum cutoff of 3 reads was used.

The Euclidean Distance (ED) algorithm was used to obtain the genetic distance to the associated QTLs. The calculation was performed as previously described [22]. Basically, the ED for each SNP was calculated using the following equation:

$$\text{ED}=\sqrt{{\left(A_{F}-A_{S}\right)}^{2}+{\left(C_{F}-C_{S}\right)}^{2}+{\left(G_{F}-G_{S}\right)}^{2}+{\left(T_{F}-T_{S}\right)}^{2}}$$

The higher the ED value is, the closer the distance is to the targeted genes. The letters represent their corresponding bases, and A_*F*_ and A_S_ represent the frequencies of A in the bulk F and bulk S, respectively. The ED values were raised to a power of 5 (ED^5^) to decrease the noise generated by small variations in the estimations. The data are fitted using a Loess curve with the fitted values as the median of the up and downstream 50 SNPs.

### Expression analysis of potential associated genes

RNA was extracted from completely sterile and partially rescued plants as described above. QPCR was conducted using 3 biological replicates each with 3 technical replicates in a 20-μl system for each potential associated genes by employing the StepOnePLUS system (SinoGene, Beijing). The 20-μl QPCR mixture contained 1 μl of diluted-cDNA, 7.5 μl of 2xSG Green PCR Master Mix, and 0.25 μl of each of the 10 μM primers. The amplification was performed as follows: 95°C for 10 min followed by 40 cycles of 95°C for 20 s; 60°C for 30 s and 72°C for 30 s. The maize Actin gene (GQ339773.1) was served as the internal control. Primers used in the QPCR are included in the S1 Table. The quantification was carried out using the relative ΔΔCt method on Prizm 5 software (http://www.graphpad.com/quickcalcs/).

### KASP assay design

Genomic DNA was extracted from 50 individual sterile plants and 50 partially rescued plants as previously described [34]. Kompetitive Alleles Specific PCR (KASP) assays were developed for 4 SNPs identified in two potential associated genes from RNA-Seq. KASP primers sequences were listed in S3 Table. KASP assays were carried out with 4 μl reaction system including 2 μl genomic DNA at 20 ng/μl, 0.106 μM of each primer and 2 μl of KASP master mix (Kbiosciences, Herts England). PCR conditions for the assays were set up as previously described [35]. PCR fluorescent endpoint readings were evaluated using the BMG Pherastar (LGC, Middlesex, UK), and the visualization of the clusters with the SNP allele callings was obtained by Kluster Caller software (LGC, Middlesex, UK).

## Results

### Identification of fertility instable plants

**Fig 1:**
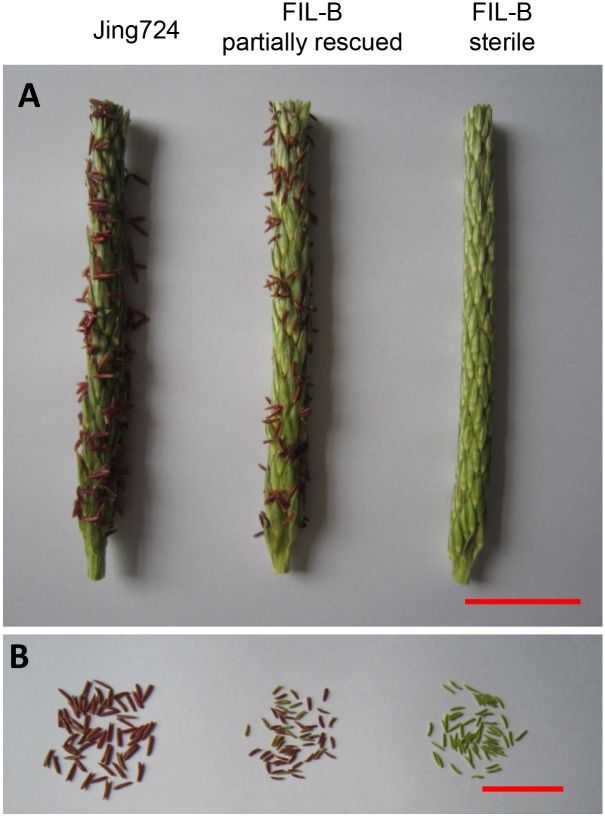
Partially restored anther exsertion in the FIL-B population. **A.** Representative image of the tassels of Jing724 and FIL-B individuals with partial anther exsertion and complete sterility at six days after tasseling. The scale bars represent 3 cm. **B.** Representative image of the anthers harvested from A. The scale bars represent 3 cm.

Anther exsertion was first observed in FIL-B population 3 days after tasseling, and 3 additional days later, approximately 1/3 of the plants showed anther exsertion (Fig 1A), which categorized the FIL-B as grade II fertility according to the classification in Feng et al.[17]. Unlike Jing724, anthers of FIL-B showed no anther dehiscence or pollen shedding (Fig 1B). The anther size of the partially rescued FIL-B was smaller than the Jing724 but was comparable to the sterile anthers. The anthers of the partially rescued plants exhibited an intermediate color and shape when compared to the Jing724 and sterile individuals. Anther exsertion served as the phenotypic classification of fertility in the BC_4_ population, which characterized 14.9% of the 462 plants as partially rescued. These data suggest that the fertility instability of FIL-B is regulated by multiple loci.

**Fig 2:**
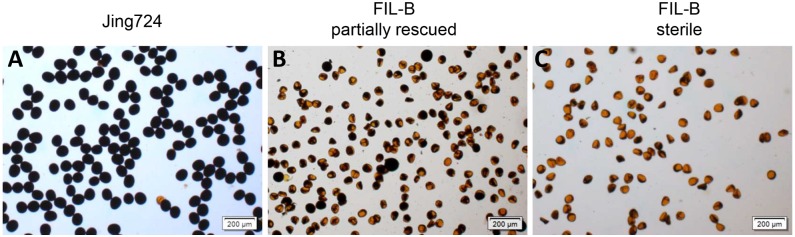
Pollen staining with I_2_-KI revealed the normal development of a small portion of pollen from the partially rescued FIL-B. Representative images of the stained pollen of Jing724 (**A**) and the partially rescued (**B**) and sterile (**C**) individuals of FIL-B. Pollen was collected when the anthers exserted in the partially rescued plants. Round pollen with black staining was recorded as normal. The scale bars represent 200 μm.

Although a small portion of pollen from the partially rescued plants showed normal development with black staining (Fig 2), the majority exhibited an irregular shape without staining, which was similar to the pollen from the sterile plants. No pollen was stained in the sterile plants, but 5.56% of the pollen from the partially rescued plants was stained to show normal starch accumulation, indicating that pollen of FIL-B is fertility instable with possible fertility restoration.

### Pollens and microspores in the partially rescued plants show partial restoration

**Fig 3:**
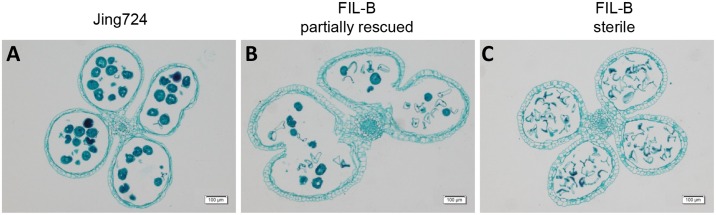
Paraffin slides show normal microspore development in the partially rescued FIL-B. Microscopic images of anther transverse sections of Jing724 (**A**) and the partially rescued (**B**) and sterile (**C**) individuals of FIL-B. The black round microspores were considered as normal. The scale bars represent 100 μm.

To test pollen development during the microspore stage in the anthers, we observed the anther dissection under a light microscope. However, the development differed in the sterile and partially rescued anthers during telophase of the uninucleate microspores (Fig 3). Although the development and structure of tapetum were the same in both genotypes, all of the microspores collapsed in the sterile anthers, while a small proportion of the microspores had normal development in the partially rescued plants. The individual normal microspores from the FIL-B partially rescued plants might lead to normal mature pollen, which is consistent with the pollen staining data and further confirms the fertility instability of FIL-B.

### Transcriptome analysis identifies 3,672 differentially expressed genes

To map the loci controlling the fertility instability in FIL-B, we performed BSR-Seq to identify the differentially expressed genes in the two bulks with distinct fertility phenotypes. Eighty-nine and 61 million clean reads were obtained for bulk F and bulk S, respectively, with an average read length of 252 bp, and the percentage of bases (Q30) over 87.09%. The clean reads were aligned to the maize reference genome B73, revealing an even distribution on the 10 chromosomes of B73, and the unique mapped reads ratio for bulk F and S were 72.79% and 69.17%, respectively. By the RNAseq, the total of 31,905 and 31,895 gene models were expressed in partially rescued and sterile bulk, respectively. We identified a total of 3,672 genes with differential expression (Fold change ≥2 and FDR<0.01) between the two bulks, among which 3,540 genes were annotated in the NR, Swiss-Prot, GO, COG and KEGG databases, and 3,548 genes were up regulated and 124 down regulated in the bulk F.

TopGO [36] analysis was performed using the Gene Ontology (GO) annotations of the genes with differential expression between the two bulks. This analysis compares the relative proportion of genes represented in the whole maize genome for specific GO terms to the proportion of same terms represented in the differentially expressed genes. This approach leads to the calculation of a KS score, which indicates the statistical significance of the enrichment for a specific GO class. We performed a TopGO analysis for all three ontologies (cellular component, molecular function and biological process), and the ten most significantly enriched terms presented with the smallest KS scores are included in S2 Table. Notably, the majority of the enriched GO categories were associated with pollen tube development (e.g., "pollen tube tip" (GO:0090404) and "pollen tube growth" (GO:0009860)), cell wall modification (e.g., "pectinesterase activity" (GO:0030599) and plant-type cell wall modification (GO:0009827)) and carbohydrate metabolism (e.g., "galacturan 1,4-alpha-galacturonidase activity" (GO:0047911) and "polygalacturonase activity" (GO:0004650)), highlighting the different expression patterns between the sterile plants and partially rescued plants. Previously, it was shown that genes involved in energy metabolism are related to pollen fertility [37, 38], indicating that the enrichment of those genes may contribute to the fertility restoration of the FIL-B in the partially rescued individuals.

To further demonstrate the over-representation of carbohydrate metabolism in the partially rescued plants, a pathway enrichment analysis was carried out for the genes with differential expression between the two bulks using the KEGG pathway database (http://www.genome.jp/kegg/). Eight pathways were significantly enriched (Table 1), and a majority of these were carbohydrate metabolism pathways with the enrichment fold ranging from 2.1 to 3.4, including "Amino sugar and nucleotide sugar metabolism" (ko00520), "Starch and sucrose metabolism" (ko00500), "Glycolysis/Gluconeogenesis" (ko00010) and "Citrate cycle" (ko00020). These results further confirmed that the overexpression of carbohydrate metabolism pathways and signal transduction pathways in the partially rescued individuals were associated to the fertile instability of FIL-B.

**Table 1:** Enrichment of the KEGG pathways in the differentially expressed genes.

Pathway	KO	Enrichment Fold	Q-value
Galactose metabolism	ko00052	3.4	1.2E-06
Amino sugar and nucleotide sugar metabolism	ko00520	2.4	6.2E-06
Glycerophospholipid metabolism	ko00564	2.6	1.5E-05
Starch and sucrose metabolism	ko00500	2.2	1.9E-05
Glycolysis / Gluconeogenesis	ko00010	2.1	3.9E-05
Pyruvate metabolism	ko00620	2.5	4.0E-05
Citrate cycle (TCA cycle)	ko00020	2.4	7.8E-05
Phosphatidylinositol signaling system	ko04070	3.2	2.2E-04

KO: KEGG pathway ID

Enrichment Fold: the ratio of the genes annotated for a specific pathway in the differentially expressed genes to the genes annotated for the same pathway in the whole genome

Q-value: P value after adjustment for multiple hypothesis testing

### Identification of six potential associated genes for fertility instability

**Fig 4:**
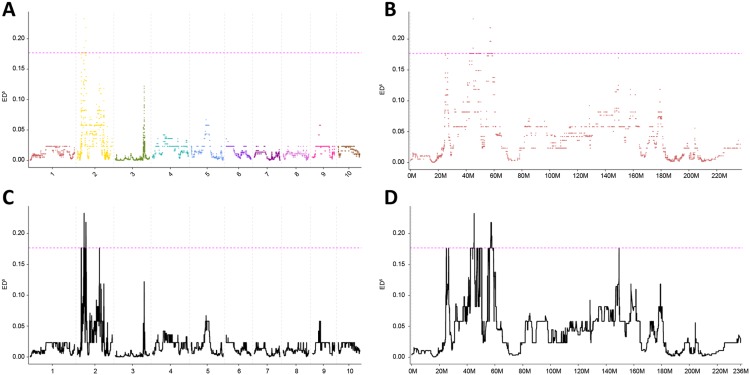
Identification of genomic regions contributing to the fertility instability in FIL-B by BSR-Seq using the Euclidean distance (ED) algorithm. **A.** The ED scores raised to the fifth power across the genome. Each dot represents each SNP identified from the RNA-Seq, and the different colors designate the different chromosomes as indicated on the X-axis. For all of the panels, the gray vertical dotted lines delineate the chromosome edges, and the width of the chromosome represents the relative numbers of SNPs identified. The pink horizontal dotted lines represent the significant threshold of the 99% percentile of the ED^5^. **B.** The ED^5^ scores of a close-up of chromosome 2. **C.** The Loess fit curve calculated from A. **D.** The Loess fit curve of a close-up of chromosome 2 with the physical position indicated on X-axis. Each peak represents a possible associated genomic region.

The sequencing reads were aligned to the B73 reference genome to obtain the SNPs in both of the bulks. A total of 171,594 SNPs and 170,820 SNPs were identified in bulk F and bulk S, respectively. To map the QTLs controlling fertility restoration, the Euclidean Distance (ED) algorithm was applied to measure allele segregation and to identify the linked genomic loci based on the SNPs between the two bulks. A statistically significant peak of ED was observed on chromosome 2 (Fig 4A and 4B), indicating its association to fertility instability in FIL-B. Seven genomic regions were associated with fertility instability by using the 99% percentile of the ED^5^ (0.17678) as the significant cutoff (Fig 4C and 4D), among which two regions consisted of only a couple of bases. The other 5 regions were all located on the short arm of chromosome 2 (Table 2). To identify the potential associated genes within these 5 genetic loci, the annotated genes were further screened based on their expression pattern within the two bulks. A total of 12 genes (Table 3) exhibited differential expression, 8 genes were located in region 2 and 4 genes were located in region 5, with no genes identified in the other 3 regions (Table 2). Of these genes, *GRMZM2G315401* and *GRMZM2G434669* encode the serine/arginine -rich protein 45, *GRMZM2G430362* encodes an ATP-dependent RNA helicase SUV3, *GRMZM2G474783* and *GRMZM2G010338* encode the leucine-rich repeat extensin-like protein 5 and a putative SPRY-domain family protein, respectively, and *GRMZM2G127173* encodes an uncoupling protein regulating ATP biosynthesis. The 6 above mentioned genes were selected as potential associated genes for the following expression study because of their possible function as regulatory genes. Moreover, *GRMZM2G012328* is predicted as pectinesterase, *GRMZM2G145758* possibly encodes histone H3, and *AC191050*.*3_FG003* encodes a potential precursor of the globulin-1 S allele, all of which may involve in cell wall metabolism and modification. The other three genes were annotated as hypothetical proteins and thus need further investigation.

**Table 2:** The physical position and number of genes in the five identified genomic regions on chromosome 2.

Region	Physical Position	Size	Gene	Differentially expressed gene
1	26,652,602–26,654,212	1.61 Kb	0	0
2	42,383,737–45,424,781	3.04 MB	85	8
3	46,980,442–47,717,638	0.74 MB	13	0
4	48,752,208–50,549,409	1.79 MB	29	0
5	55,113,525–58,997,475	3.88 MB	63	4

**Table 3:** Twelve genes with differential expression from the 5 genomic regions identified on chromosome 2.

Gene ID	Annotation	FDR	Fold Change	Regulated
*GRMZM2G010338*	Putative SPRY-domain family protein	8.97E-09	10.3	up
*GRMZM2G012328*	Pectinesterase	1.35E-12	19.5	up
*GRMZM2G127173*	Mitochondrial uncoupling protein 3	0.00053	4.6	up
*GRMZM2G145758*	Histone H3	9.40E-05	5.6	up
*GRMZM2G315401*	Serine/arginine-rich protein 45	7.96E-10	11.9	up
*GRMZM2G347489*	Hypothetical protein	2.85E-11	16.7	up
*GRMZM2G430362*	ATP-dependent RNA helicase SUV3	1.02E-06	7.4	up
*GRMZM2G474783*	Leucine-rich repeat extensin-like protein 5	5.52E-11	14.4	up
*AC191050*.*3_FG003*	Globulin-1 S allele	0.00073	5.8	up
*GRMZM2G406026*	Hypothetical protein	3.07E-12	18.0	up
*GRMZM2G434669*	Serine/arginine -rich protein 45	0.001	4.3	up
*GRMZM5G858609*	Hypothetical protein	2.63E-07	9.2	up

### Expression analysis of the potential associated genes validates the difference between the two bulks

**Fig 5:**
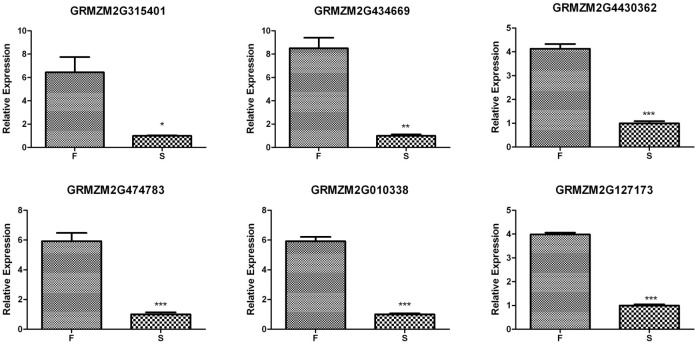
Comparison of the transcript levels of the six potential associated genes from the partially rescued (F) and sterile (S) plants of FIL-B, as detected by QPCR. Each bar represents the mean±SE of the biological replicates. The values are calculated using Actin as an internal control. The asterisks show the statistically significant difference compared to the partially rescued plants, as determined by the analysis of variance: * (P<0.05), **(P<0.01), ***(P<0.001).

QPCR was performed for the six genes using individual plants to validate the RNA-Seq data and to obtain more quantitative transcript level measurements. The relative expression of all of the six potential associated genes showed a 4- to 8-fold up regulation in the partially rescued plants compared to the sterile plants (Fig 5). The trends in regulation exhibited a consistent pattern between methods and thus, confirming the qualitative values of the RNA-Seq.

### Validation of SNPs identified from RNA-Seq

**Fig 6:**
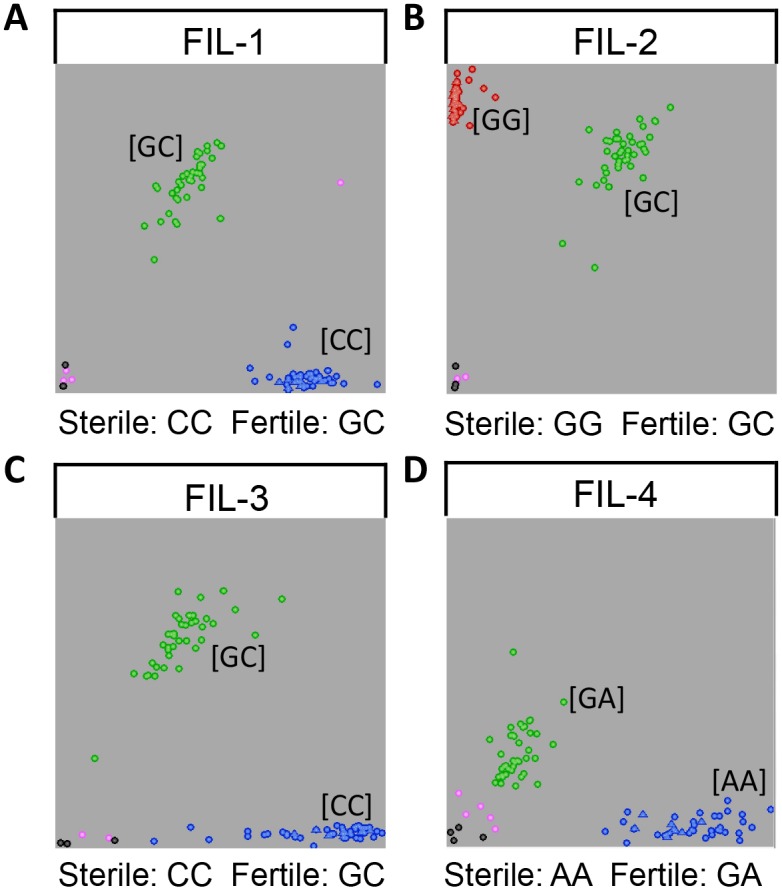
Graphs of four KASP marker assays. KASP assays were developed for SNPs identified in two potential associated genes from RNA-Seq. Fertility instable line (FIL)-1 and FIL-2 were SNPs resided in *GRMZM2G315401*. FIL-3 and FIL-4 were SNPs detected in *GRMZM2G430362*. The triangles represent the recurrent parent of Jing724. Black data points are negative control and pink ones are ambiguous calling.

To further assess the effect of the potential associated genes, SNPs identified in six potential associated genes were evaluated. Two SNPs in each of the *GRMZM2G315401* and *GRMZM2G430362* appeared to be conclusive, whose alleles and positions on B73 were included in Table 4. To further determine the correlation between these SNPs and the fertility phenotype, KASP marker assays were performed with 50 individuals of sterile plant and 50 partially rescued plants from the BC_4_ population. As expected, all the sterile individuals possessed the homozygous loci, and all the fertile plants exhibited heterozygous loci for all four SNPs with no exceptions (Table 4 and Fig 6). The PCR-based SNP assays validated the SNPs identified from RNA-Seq, further confirming the potential function of two associated genes in fertility instability.

**Table 4:** SNPs in genes potentially associated with fertility instability identified from RNA-Seq

KASP Assay	Gene	Alt	Allele of recurrent parent Jing724	SNP positions
FIL-1	*GRMZM2G315401*	C/G	C	42994131
FIL-2	*GRMZM2G315401*	G/C	G	42995108
FIL-3	*GRMZM2G430362*	C/G	C	42743133
FIL-4	*GRMZM2G430362*	A/G	A	42743749

Alt: allele in sterile plant/ allele in fertile plant

SNP positions: physical positions of SNP on B73 genome

## Discussion

CMS is widely exploited in maize hybrid breeding to avoid excessive labor for artificial emasculation and possible hybrid contaminations. Therefore, tremendous efforts are made to understand the molecular mechanism of CMS-S in maize [9, 11]. The maize cytoplasmic gene *orf77* controls male sterility in CMS-S and contains three *atp9* chimeric sequences, which have affected the function of ATP9 [11]. Due to the importance of ATP9 in the energy metabolism of the mitochondria, the chimeric sequences are considered to lead to pollen sterility [3]. The partial fertility restoration in FIL-B suggests that multiple minor effect loci may contribute to the process, which eliminates the energy loss due to sterile gene expression. CMS-S in maize is gametophytic male sterility, whose fertility is conditioned by the pollen genotype instead of its maternal genetic background [39]. Therefore, we select anther tissues as study materials, where the formation and development of pollen occur. In this study, we revealed over 3,500 differentially expressed genes between the two bulks of FIL-B, each bulk consisted of 30 individual plants based on the anther exsertion phenotypes. The differentially expressed genes were categorized as part of the starch and sucrose metabolism pathway, the gluconeogenesis pathway, the TCA pathway, etc. (Table 1). In addition, an overrepresentation of the genes involved in cell wall modification, pollen tube growth and regulatory enzymatic activity was also observed (S2 Table). Using BSR-Seq, we located the minor effect QTLs to 5 genomic regions on the short arm of chromosome 2 (Fig 4). The five genomic regions identified in this study are not consistent with the previous study of fertility instability of CMS-S [17, 18]. The discrepancy is probably due to the different methods and different mapping population used, which further suggests that fertility instability is conditioned by many minor effect loci.

Among the 12 differentially expressed genes within these 5 genomic regions, 6 were identified as potential associated genes with regulatory annotations. The expression level of these 6 genes were confirmed with QPCR (Fig 5), and the four SNPs of two potential associated genes identified from RNA-Seq were validated with KASP marker assays (Fig 6). Two of the potential associated genes, *GRMZM2G315401* and *GRMZM2G434669*, were annotated as Serine/arginine-rich protein 45 (SR45), which plays a crucial role in constitutive and alternative mRNA splicing and metabolism [40, 41]. SR45 is involved in floral organ morphogenesis and carbohydrate metabolism in Arabidopsis [42, 43]. *GRMZM2G474783* was annotated as a Leucine-rich repeat (LRR) extensin-like protein 5, and the LRR motif is believed to bind to a specific ligand during pollen tube development in tomato [44]. *GRMZM2G010338* was predicted to be a putative SPRY-domain family protein, which is considered to be involved in protein-protein interactions. By combining the corresponding receptors, the SPRY structure domain participates in the cytokine signaling pathway through protein ubiquination and degradation in primates [45, 46]. These genes may regulate pollen starch accumulation, floral organ development, carbohydrate metabolism and other related biological process, resulting in fertility restoration with anther exsertion in the partially rescued plants. In addition, *GRMZM2G430362* encodes an ATP-dependent RNA helicase SUV3, a key control element in nuclear-mitochondrial interactions, whose function has been implicated in a variety of mitochondrial posttranscriptional and translational processes in yeast and some plants [47–50]. The higher expression of this gene in the partially rescued plants indicates its possible modification of the cytoplasmic sterility factor, leading to the restoration of pollen fertility.

Interestingly, *GRMZM2G127173* encodes a mitochondrial uncoupling protein 3 (UCP3), which is a mitochondrial transporter located in the inner membrane of the mitochondria, and this protein was also up regulated in the partially rescued plants. Pollen maturation requires a great amount of energy, and it has been proposed that the cytoplasmic sterility gene compromises the electron transport chain of the mitochondria, which is then unable to provide sufficient ATP for normal pollen development in beets and rice [38, 51]. Previous research has demonstrated that the UCPs reduce the proton potential across the mitochondrial inner membrane and eliminate ATP biosynthesis in plants and mammals [52–54]. It is possible that the over expression of UCP3 in the partially rescued plants serves as a negative feedback mechanism. Thus, unlike the direct inhibition of mitochondrial *orf355-orf77* expression by the major effect loci of *Rf3*, the extent of anther exsertion and the percentage of fertility restoration in FIL-B are resulted from a fine-tuned coordination of a set of minor effect loci and their interactions.

Unlike the major restorer *Rf3*, the contributions of minor effect loci are susceptible to environmental factors. Temperature, humidity and total transpiration change the fertility stability of CMS-S [8, 55]. A significantly different ratio of fertile restoration was observed in FIL-B at two locations with a temperature difference of 3–5°C (data not shown), and the complicated interaction with the environment made the illustration of the mechanism a tedious topic. Despite of the fertility restoration in FIL-B, some of the families selected from the backcross population between MD32 and Jing724 were stably sterile, which were already successfully applied in the commercial seed production of Jingke968 [25].

Based on the study of the fertility instability of FIL-B, we conclude that (1) multiple minor effect loci are involved in the partial fertility restoration, especially genes functioning in the regulation of floral organ differentiation and development, energy metabolism and carbohydrates biosynthesis; (2) the regulatory mechanism of pollen sterility is considerably complicated, and the fertility instability phenotype in a specific nuclear background may result from the interaction of positive and negative effect minor genes; and (3) minor effect fertility restoration genes are easily affected by environmental factors.

The identification of genes potentially associated with fertility instability in this study sheds light on the theoretical knowledge of its mechanism, which could facilitate the commercial promotion of CMS-S in maize breeding. Future investigation is needed to elucidate their functions and possible interactions at the molecular level.

## Supporting Information

S1 Dataset Raw RNA-Seq data: http://pan.baidu.com/s/1jIGp5Qm. (DOCX)

S1 Table Primers used for QPCR. (XLSX)

S2 Table Top 10 most significantly enriched GO classes for each ontology group by TopGO analysis. (XLSX)

S3 Table Primer sequences used for KASP assays. (XLSX)

S4 Table SSR fingerprint information of the Jing724 backcross progeny. (XLSX)
