# Supplementary material for: Identification of Genes Potentially Associated with the Fertility Instability of S-Type Cytoplasmic Male Sterility in Maize via Bulked Segregant RNA-Seq
Source: PLoS One. 2016 Sep 26;11(9):e0163489. doi: 10.1371/journal.pone.0163489 (PMC5036866; doi:10.1371/journal.pone.0163489)
Supplement: S1 Dataset — (DOCX) [file pone.0163489.s001.docx]

S1 Dataset. Raw RNA-Seq data

http://pan.baidu.com/s/1jIGp5Qm
